# Supplementary material for: An artificial intelligence-based model for optimal conjunctive operation of surface and groundwater resources
Source: Nat Commun. 2024 Jan 16;15:553. doi: 10.1038/s41467-024-44758-6 (PMC10791678; doi:10.1038/s41467-024-44758-6)
Supplement: Supplementary file 1 — Supplementary Information [file 41467_2024_44758_MOESM1_ESM.pdf]

Supplementary Information

An Artificial Intelligence-Based Model for Optimal Conjunctive Operation of Surface and Groundwater Resources

Saeid Akbarifard[1,2*], Mohamad Reza Madadi[3*], Mohammad Zounemat-Kermani[4*]

1. *Department of Water Engineering, Faculty of Civil and Surveying Engineering, Graduate University of Advanced Technology, P.O. Box 76315116, Kerman, Iran*
2. *Research and Technology Institute of Plant Production, Shahid Bahonar University of Kerman, Kerman, Iran. (*Corresponding Author: akbarifard_saeid@agr.uk.ac.ir), ORCID: 0000-0003-0965-4329.*
3. *Department of Water Engineering, Faculty of Agriculture, University of Jiroft, Jiroft, Iran (*Corresponding Author: madadi@ujiroft.ac.ir), ORCID: 0000-0002-6734-7944.*
4. *Department of Water Engineering, Faculty of Agriculture, Shahid Bahonar University of Kerman, Kerman, Iran. (*Corresponding Author: zounemat@uk.ac.ir), ORCID: 0000-0002-1421-8671.*

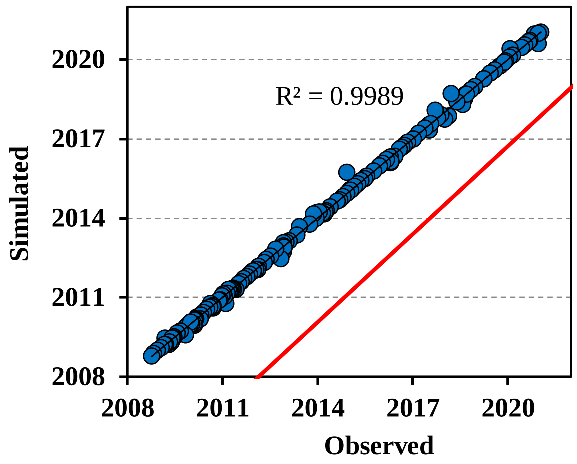

(a)

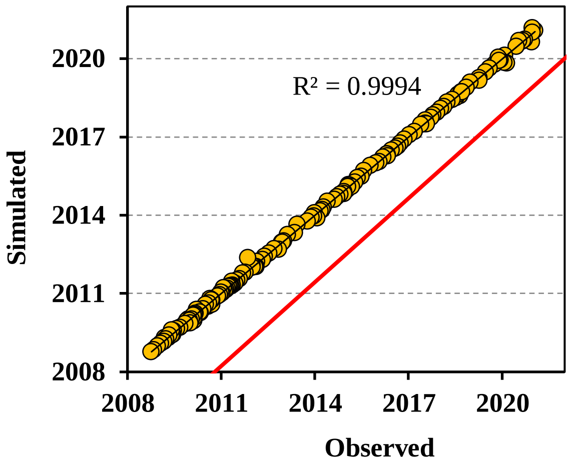

(b)

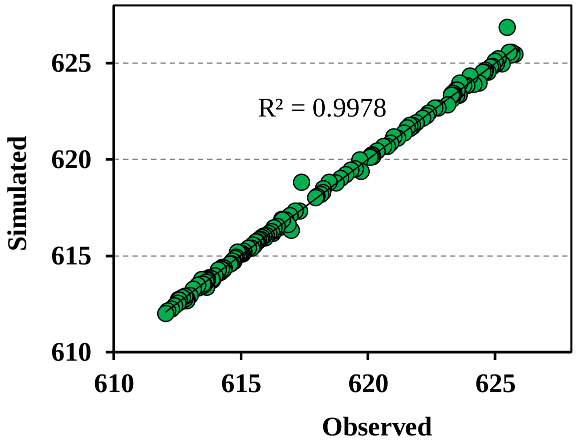

(c)

Supplementary Figure 1. Observed groundwater level versus the simulated values by arttificial neural network (ANN) model in (**a**) Baft (**b**) Rabor (**c**) Jiroft (the redline shows the 45-degree line)

Here, the higher the concentration of points around the 45-degree line, the higher the accuracy of the simulations. The values of determination cofficient (R^2) is very close to 1, which means that the ANN simulation model has performed extremely well in simulating groundwater level.

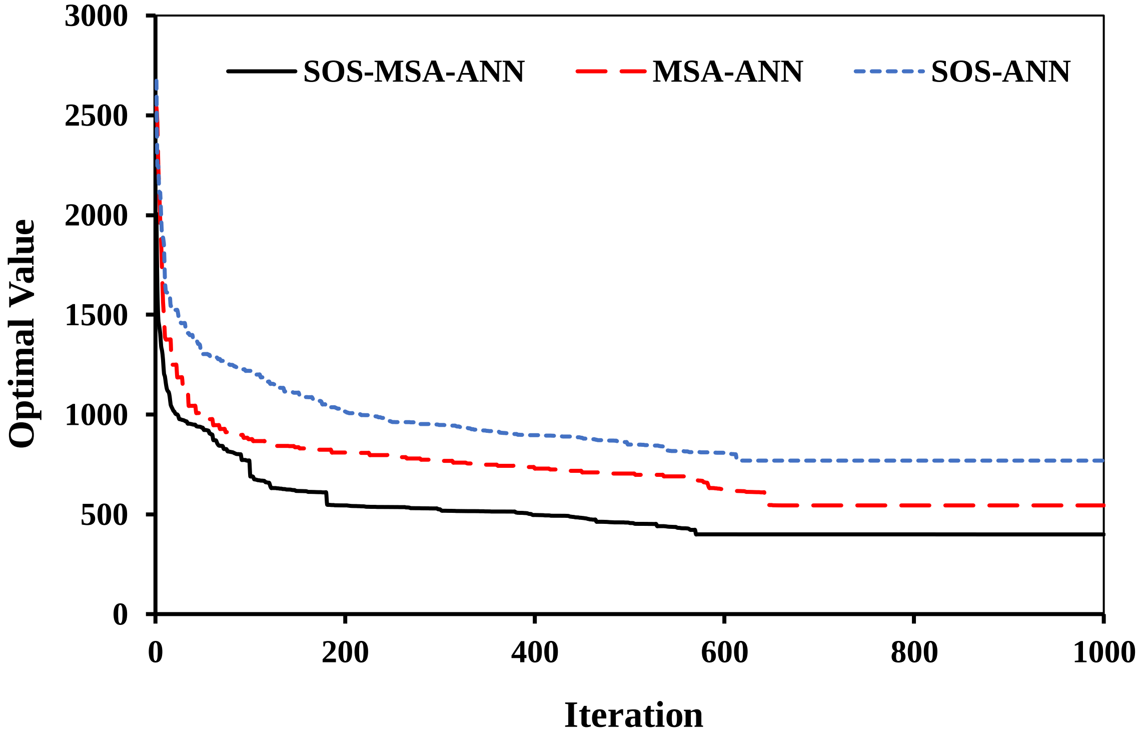

Supplementary Figure 2. Convergence rate to the optimal value in the problem of conjunctive operation of surface and groundwater resources in Halilrood basin for the developed models

Here, the **SOS-MSA-ANN** is the hybrid simulation-optimization model developed in this study. It was obtained by the combination of symbiotic organism search algorithm and moth swarm algorithm with the artificial neural network. Similarly, the MSA-ANN and SOS-ANN are the other two first-level simulation-optimization models developed in this study. By increasing the number of iterations, the solutions produced by the models get closer to the optimal value. A more successful model is one that can produce optimal solutions in fewer iterations.

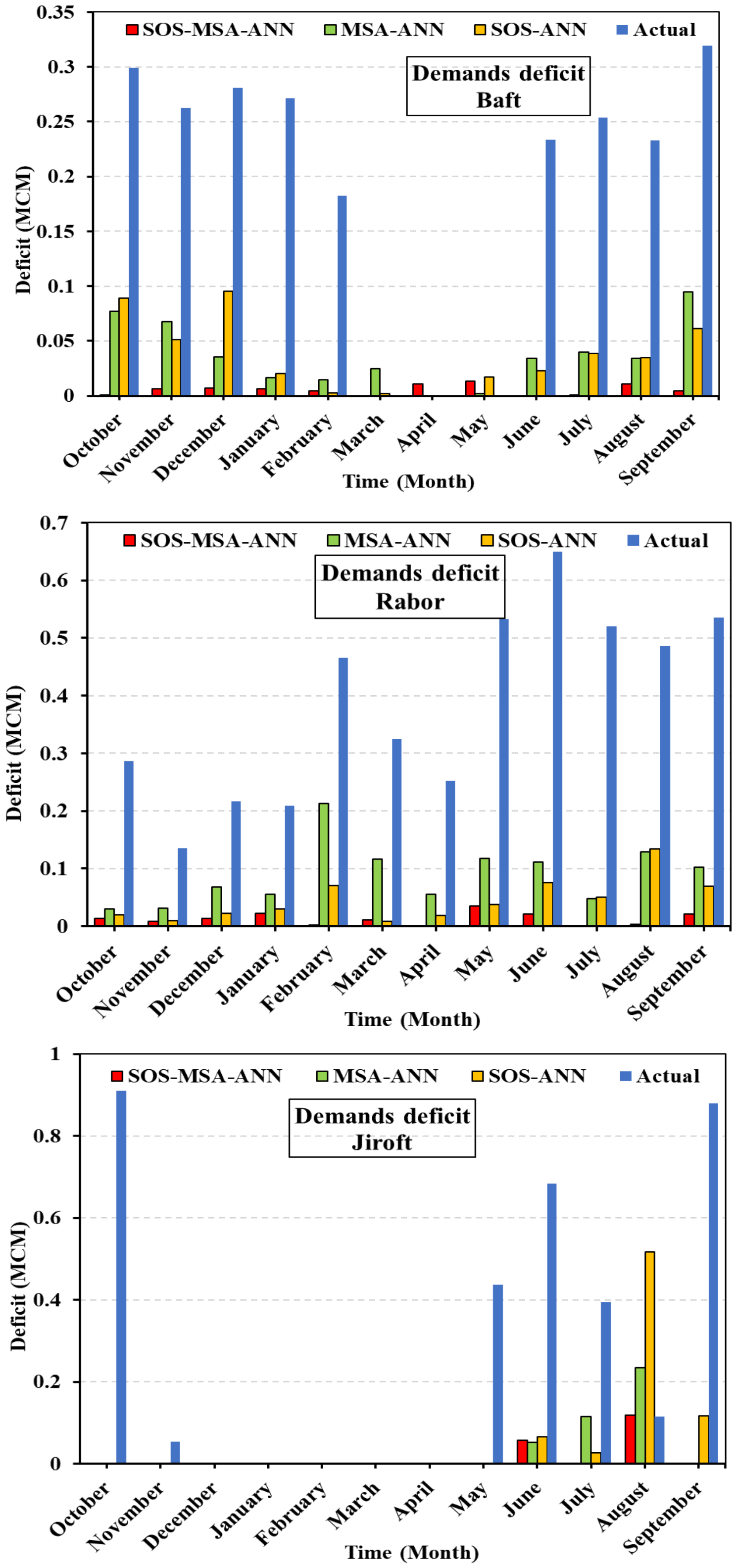

Supplementary Figure 3. Monthly average deficit resulting from operation scenarios of the developed models in the three regions of the Halilrood basin (study period: 2001-2019)

Here, the values of demands deficit in real condition are compared with the corresponding values obtained by the developed models. The smaller the height of the histogram, the better the performance of the corresponding model. The best case is when the height of the histogram is zero, this means that in those months, no deficit has been observed i.e., the total demand has been met.

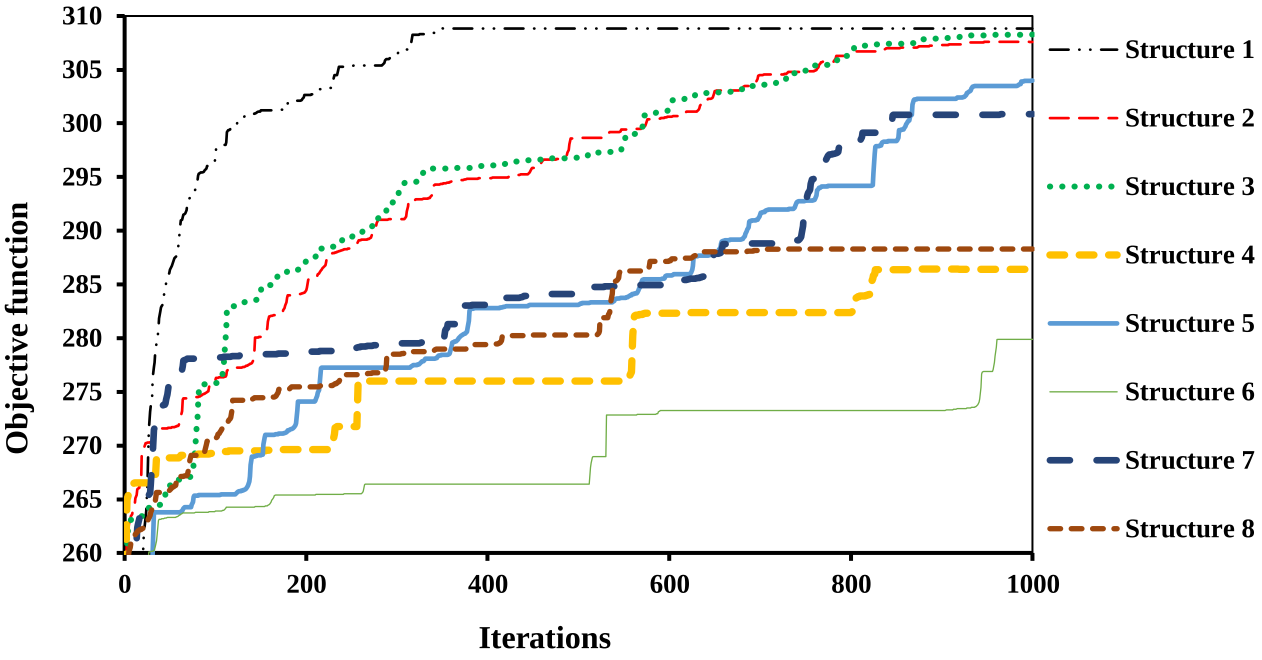

Supplementary Figure 4. Comparing the convergence rate to the optimal value in eight MSA structures for the benchmark problem

Here, 8 different structures for the moth swarm algorithm (MSA) were investigated. For each structure, the convergence rate of the MSA was calculated. A structure that can produce solutions closer to the optimal solution (objective function value) in less number of iterations is considered the best structure.

Supplementary Table 1. Utilized data range in the study area (the values represent average annual quantities)

Regions	Data range	Precipitation (mm)	Evaporation (mm)	Average river inflow (MCM)	Groundwater level (MASL)	Total water demands (MCM)
**Baft**	**Minimum**	0	0	0.13	2009.62	0.61
	**Mean**	20.05	206.33	1.64	2016.88	1.45
	**Maximum**	273.6	488.5	35.4	2024.5	2.9
**Rabor**	**Minimum**	0	0	0	2008.77	1.05
	**Mean**	19.046	61.71	2.073	2014.94	2.54
	**Maximum**	271	488.5	42.06	2022.32	4.77
**Jiroft**	**Minimum**	0	0	0.25	612.05	3.18
	**Mean**	13.03	257.59	14.09	619.35	5.3
	**Maximum**	125.3	558.5	407.91	628.11	7.65

Supplementary Table 2. Parameter setting values for simulation- optimization model

Methods	Parameters	Values
# For all optimization models	Maximum iterations	1000
	Number of variables	1338
MSA	Number of search agents	500
	Number of pathfinders	50
SOS	Ecosystem population size	500
	BF1	1 or 2
	BF2	1 or 2
ANN	Hidden layers' structure	10-15-10
	Hidden layers transfer function	RBF
	Output layer transfer function	Purelin
	Performance function	MSE
	Training function	Levenberg-Marquardt
