# Supplementary material for: An artificial intelligence-based model for optimal conjunctive operation of surface and groundwater resources
Source: Nat Commun. 2024 Jan 16;15:553. doi: 10.1038/s41467-024-44758-6 (PMC10791678; doi:10.1038/s41467-024-44758-6)
Supplement: Supplementary file 3 — Description of Additional Supplementary Files [file 41467_2024_44758_MOESM3_ESM.pdf]

Description of Additional Supplementary Files

File Name: Supplementary Code 1
Description: Simulation-optimization model scripted in programming panel of MATLAB software. It contains the main code (main_MSA_SOS_hybrid.m) and respective subroutines.
