# Supplementary material for: An artificial intelligence-based model for optimal conjunctive operation of surface and groundwater resources
Source: Nat Commun. 2024 Jan 16;15:553. doi: 10.1038/s41467-024-44758-6 (PMC10791678; doi:10.1038/s41467-024-44758-6)
Supplement: Supplementary file 4 — Supplementary Code 1 [file 41467_2024_44758_MOESM4_ESM.zip › Supplementary Code 1/Read Me.pdf]

Here the “**main_MSA_SOS_hybrid.m**” is the main code of developed simulation-optimization model that was scripted in programming panel of MATLAB software. The other 8 codes are the sub-routines for the main code. When the main code is run, the subroutines, each with their own specific tasks, are also executed. All these subroutines in connection with the main code form the main simulation-optimization model:

1. **SOS_MSA.m**: this is the developed hybrid MSA-SOS optimization algorithm.

2. **SOS_Hybrid.m**: this is the developed SOS algorithm which is added to the hybrid algorithm.

3. **initialization.m**: this is related to the initialization of the hybrid optimization algorithm.

4. **Bound_Checking.m:** this is related to the limitations and boundary conditions of the algorithm.

5. **Objective_Function_details_Simulation_Model.m**: this is related to the objective function and constraints of the problem.

6. **ANN_Baft.m:** this is related to the developed ANN simulator for estimating the groundwater level in the Baft region.

7. **ANN_Jiroft.m:** this is related to the developed ANN simulator for estimating the groundwater level in the Jiroft region.

8. **ANN_Rabor.m**: this is related to the developed ANN simulator for estimating the groundwater level in the Rabor region.
